# A rising tide lifts all boats: The social contagion of achievement in L2 classrooms and the role of intrinsic motivation and engagement

**DOI:** 10.1111/bjep.12752

**Published:** 2025-02-25

**Authors:** Norman B. Mendoza, Artem Zadorozhnyy, John Ian Wilzon T. Dizon

**Affiliations:** ^1^ Department of Curriculum and Instruction The Education University of Hong Kong Hong Kong SAR China; ^2^ Department of English Language Education The Education University of Hong Kong Hong Kong SAR China; ^3^ Bau Institute of Medical and Health Sciences Education The University of Hong Kong Hong Kong SAR China

**Keywords:** achievement composition effect, engagement, intrinsic motivation, L2 learning

## Abstract

**Background:**

The achievement composition effect (ACE) posits that students' academic performance is influenced by the collective achievement level of their classmates. While ACE has been demonstrated across various learning domains, its role in second language (L2) learning and motivational moderators of this effect remain underexplored.

**Aims:**

This longitudinal study examines ACE in the context of L2 learning, with a particular focus on the moderating roles of students' intrinsic motivation and engagement.

**Methods:**

A sample of 766 secondary school L2 learners from 30 classrooms was analysed using linear mixed‐effects models to investigate the relationship between students' relative achievement at Time 1 and their subsequent achievement at Time 2, as well as the moderating effects of intrinsic motivation (to know, to accomplish and to experience stimulation) and engagement (behavioural and emotional).

**Results:**

Results indicate that students' relative achievement significantly predicts subsequent achievement, supporting the presence of ACE in L2 classrooms. Moreover, intrinsic motivation to experience stimulation, behavioural engagement and emotional engagement significantly moderated this relationship in that ACE was stronger among students with higher levels of these motivational and engagement factors.

**Conclusion:**

The findings highlight the interplay between peer achievement and individual motivational factors in shaping learning outcomes. The discussion situates these results within the broader literature on peer influence, motivation and engagement, exploring their theoretical and practical implications for L2 learning. The study underscores the importance of considering social, motivational, affective and behavioural factors in understanding and fostering optimal L2 learning environments.

## INTRODUCTION

Peer relationships play a crucial role in shaping students' academic achievement (Kilday & Ryan, 2022; Steenberghs et al., 2021). One way in which peers influence academic outcomes is through the achievement composition effect (ACE), which posits that students' academic performance is influenced by the collective achievement level of their classmates (Becker et al., 2022; Fortuin et al., 2015). This contextual effect on achievement has been extensively researched, with studies examining its impact on various academic outcomes across different educational contexts (Burns & Mason, 2002; Hanushek et al., 2003; Marsh et al., 2023; Nomi & Raudenbush, 2016; Wagner, 2022; Willms, 1985). While the role of ACE has been highlighted across academic domains (Becker et al., 2022; Hanushek et al., 2003; Marsh et al., 2023), its role in second language (L2) learning and the potential moderators of this effect remain underexplored. This research trajectory is especially crucial given that L2 acquisition is fundamentally social, communicative, and interactive in nature (Dörnyei & Ryan, 2015; Ellis, 2015), where peer interactions impact knowledge co‐construction (Sato & Ballinger, 2016). Therefore, drawing on Self‐Determination Theory (SDT; Ryan & Deci, 2017) and the self‐system model of motivational development (SSMMD; Connell & Wellborn, 1991; Skinner, Kindermann, Connell, & Wellborn, 2009; Skinner, Kindermann, & Furrer, 2009; Skinner et al., 2022), this longitudinal study examines ACE in the context of L2 learning, with a particular focus on the moderating roles of students' intrinsic motivation and engagement.

### Understanding peer effects through the lens of self‐determination theory

Two motivational frameworks, Self‐Determination Theory (SDT) and the self‐system model of motivational development (SSMMD), posit that individuals have innate psychological needs for autonomy, competence, and relatedness, the satisfaction of which enhances motivation, engagement, and overall well‐being (Deci & Ryan, 1985; Dincer et al., 2019; Ryan & Deci, 2000, 2017). While SDT emphasizes the role of supportive environments in fulfilling these needs, SSMMD mainly focuses on how individuals' self‐appraisals of their basic psychological needs impact their engagement and growth in a particular domain (Skinner, Kindermann, Connell, & Wellborn, 2009; Skinner, Kindermann, & Furrer, 2009; Skinner et al., 2022; e.g., Mendoza et al., 2022). In educational contexts, need‐supportive environments that provide opportunities for choice, optimal challenge, and a sense of belonging can facilitate students' autonomous motivation and engagement (Jang et al., 2010; Reeve & Jang, 2006; Reeve & Lee, 2014; Skinner et al., 2008; Skinner, Kindermann, Connell, & Wellborn, 2009; Skinner, Kindermann, & Furrer, 2009). Meta‐analytic studies show that peer effects can satisfy basic psychological needs through supportive classroom environments, which can foster autonomous motivation and engagement (see Ryan et al., 2022; Vasconcellos et al., 2020), potentially influencing students' receptivity to positive peer influences (Burgess et al., 2018; Laninga‐Wijnen et al., 2018).

Peer effects play an important role in shaping students' achievement and learning outcomes (e.g., Gottfried, 2014; Steenberghs et al., 2021), as the social ecology of the classroom provides opportunities for peer observation, modelling, and satisfaction of basic psychological needs (Bandura, 1997; Ryan & Deci, 2000). Empirical evidence supports these theoretical propositions, demonstrating that the presence of high‐achieving peers can exert considerable influence on students' achievement (Becker et al., 2022; Cooley Fruehwirth, 2013; Hanushek et al., 2003; Laninga‐Wijnen et al., 2018). This aligns with the idea that achievement corresponds to students' social contexts (Eccles & Wigfield, 2020; Schunk & DiBenedetto, 2020). This contextual effect, known as achievement composition effect, has been extensively researched, with studies examining its impact on various academic outcomes across different educational contexts (Burns & Mason, 2002; Hanushek et al., 2003; Marsh et al., 2023; Nomi & Raudenbush, 2016; Wagner, 2022). Specifically, this effect refers to the influence of classroom‐level achievement on individual student outcomes, above and beyond the effects of individual characteristics (Becker et al., 2022). It posits that a student's academic performance is influenced by the collective achievement of their peers.

In L2 learning, understanding the role of classroom achievement composition is crucial, as it could reveal how peer effects shape language acquisition and development. Language learning process is influenced by various individual and contextual factors (Dörnyei & Ryan, 2015; Ortega, 2009), and peer interactions are important in providing opportunities for language practice, feedback, socialization and co‐construction of knowledge (Philp et al., 2014; Sato & Ballinger, 2016). Therefore, the achievement levels of classmates may have a more direct and immediate impact on an individual's language development compared to other subjects where learning is more individualized or content‐focused. However, despite some studies on peer effects on L2 motivation and engagement (Kozaki & Ross, 2011; Peng, 2019), research on the impact of classroom achievement composition on individual L2 achievement remains scarce. This is crucial in extending the understanding of the centrality of students' social environments, including classroom effects and their impact on achievement (Daumiller & Hemi, 2024; Getenet, 2023; Kilday & Ryan, 2022; Vu et al., 2022). Moreover, few studies have looked into the role of individual‐level moderators, such as motivation and engagement, in shaping the impact of achievement composition on L2 learning. Given this, our study aims to recognize and investigate the critical roles of both the external environment—specifically classroom achievement composition—and individual‐level moderators such as motivation and engagement in shaping L2 learning outcomes.

### Achievement composition effect in L2 classrooms

Second language learning is a complex process that involves not only cognitive factors but also social and affective dimensions (Dörnyei & Ryan, 2015; Ortega, 2009). Peer interactions play a vital role in L2 learning, such as providing opportunities for authentic language use, negotiation of meaning, and socialization into the target language community (Philp et al., 2014; Sato & Ballinger, 2016). The social context of the L2 classroom can influence students' motivation, engagement, and willingness to communicate in the target language (Cao, 2011; Khajavy et al., 2018; Peng, 2019).

Recent studies have focused on how classroom‐level achievement impacts student‐level achievement (Becker et al., 2022; Fortuin et al., 2015), with evidence showing that class achievement is positively associated with individual achievement (Becker et al., 2022) and academic self‐concept (Stäbler et al., 2017). Burgess et al. (2018) highlight that peer relationships can shape students' academic motivation through processes such as social learning, need satisfaction, and the internalization of classroom norms. For example, being surrounded by highly motivated and achieving peers may enhance students' sense of competence, autonomy and belonging, leading to greater engagement and achievement in L2 learning (Ryan & Deci, 2017).

Still, to date, the impact of ACE on L2 learning outcomes remains unexamined. While some studies have investigated peer effects on L2 motivation and engagement (Kozaki & Ross, 2011; Peng, 2019; Rjosk et al., 2017), research on how these individual‐level factors can amplify or attenuate ACE in L2 classrooms remains scarce. Therefore, examining the moderating roles of intrinsic motivation and engagement in ACE within L2 classrooms can provide valuable insights into the social dynamics that drive language acquisition and academic success.

### The moderating role of intrinsic motivation and engagement in ACE

Intrinsic motivation and engagement, key constructs within Self‐Determination Theory (SDT; Ryan & Deci, 2017) and the self‐system model of motivational development (SSMMD; Connell & Wellborn, 1991; Skinner, Kindermann, Connell, & Wellborn, 2009; Skinner, Kindermann, & Furrer, 2009), respectively, may moderate the strength of the achievement composition effect (ACE) in L2 classrooms. These motivational factors have been consistently linked to positive academic outcomes, such as enhanced learning, persistence, and well‐being (Jang et al., 2012; Jang et al., 2019; Reeve & Lee, 2014; Ryan & Deci, 2020; Skinner, Kindermann, Connell, & Wellborn, 2009; Skinner, Kindermann, & Furrer, 2009).

Intrinsic motivation, the inherent satisfaction and enjoyment from engaging in an activity itself (Ryan & Deci, 2000), can be differentiated into three types: intrinsic motivation to know, to accomplish, and to experience stimulation (Vallerand et al., 1992, 1993). These distinctions have been empirically supported and have shown differential relationships with various educational outcomes (Carbonneau et al., 2012; Noels et al., 2000). Engagement, on the other hand, encompasses behavioural and emotional dimensions (Fredricks et al., 2004; Oga‐Baldwin & Nakata, 2017; Skinner, Kindermann, Connell, & Wellborn, 2009; Skinner, Kindermann, & Furrer, 2009). Behavioural engagement refers to students' active participation, effort, and persistence in learning activities, while emotional engagement involves students' positive affective reactions, such as interest, enjoyment, and enthusiasm towards learning tasks and the classroom environment. These dimensions of engagement have been found to predict academic achievement and overall school success (Christenson et al., 2012; Reeve & Tseng, 2011; Skinner & Pitzer, 2012).

We propose that intrinsic motivation and engagement may strengthen ACE in L2 classrooms by influencing students' receptivity to peer influences and their internalization of classroom norms. Highly intrinsically motivated students may be more attuned to high‐achieving peers' learning behaviours and strategies (Burgess et al., 2018; Fink & Wild, 1995) and more likely to engage in observational learning and peer modelling (Bandura, 1986). This can contribute to the contagion of achievement‐related behaviours and attitudes (Parr & Townsend, 2002; Radel et al., 2010; Ryan, 2001). They may internalize the classroom norms and values of high‐achieving peers (Ryan & Deci, 2017), perceiving their peers' success as congruent with their own interests and values (Deci et al., 1991; Wentzel & Caldwell, 1997). This internalization process may be particularly salient for students with a strong intrinsic motivation to know and accomplish, as they derive satisfaction from mastering new skills (Vallerand et al., 1992, 1993). Thus, higher intrinsic motivation may amplify the positive influence of high‐achieving peers on individual achievement, strengthening ACE in L2 classrooms.

Similarly, behaviourally and emotionally engaged students may be more responsive to peer cues and more involved in peer learning processes. Behaviourally engaged students in classroom activities have more opportunities to learn from their high‐achieving peers (Gremmen et al., 2017; Wentzel & Caldwell, 1997), facilitating the exchange of knowledge and strategies, and enhancing the contagion of achievement‐related behaviours (Burgess et al., 2018; Kindermann, 2007; Rambaran et al., 2017). Emotionally engaged students, who experience positive affect and enjoyment in learning, are more receptive to the motivational cues and learning opportunities provided by their peers (Eccles & Roeser, 2011; Furrer & Skinner, 2003; Skinner, Kindermann, Connell, & Wellborn, 2009; Skinner, Kindermann, & Furrer, 2009), and are more likely to emulate the learning practices and attitudes of their high‐achieving classmates (Ryan & Deci, 2017; Wentzel, 2009). Thus, higher levels of behavioural and emotional engagement may strengthen the impact of peer achievement on individual achievement, amplifying the ACE in L2 classrooms.

While the moderating roles of intrinsic motivation and engagement in ACE have yet to be empirically investigated in L2 settings, there is evidence from other educational domains that supports our hypotheses. For instance, studies have shown that students with higher intrinsic motivation could benefit more from cooperative learning and peer tutoring (Ginsburg‐Block et al., 2006; Roth, 2008), and those with higher engagement are more susceptible to the influence of their peers' academic behaviours and attitudes (Blansky et al., 2013; Eccles & Roeser, 2011; Juvonen et al., 2012; Shin & Ryan, 2014). These findings posit that intrinsic motivation and engagement may increase the strength of ACE on individual achievement.

### The present study

This study aims to address the gaps in the literature by examining the role of ACE in L2 learning and the moderating effects of intrinsic motivation and engagement on this relationship. In doing so, we investigate how students' relative achievement within L2 classrooms predicts their subsequent achievement, and whether this relationship is moderated by the levels of intrinsic motivation (to know, to accomplish, and to experience stimulation) and engagement (behavioural and emotional).

**FIGURE 1:**
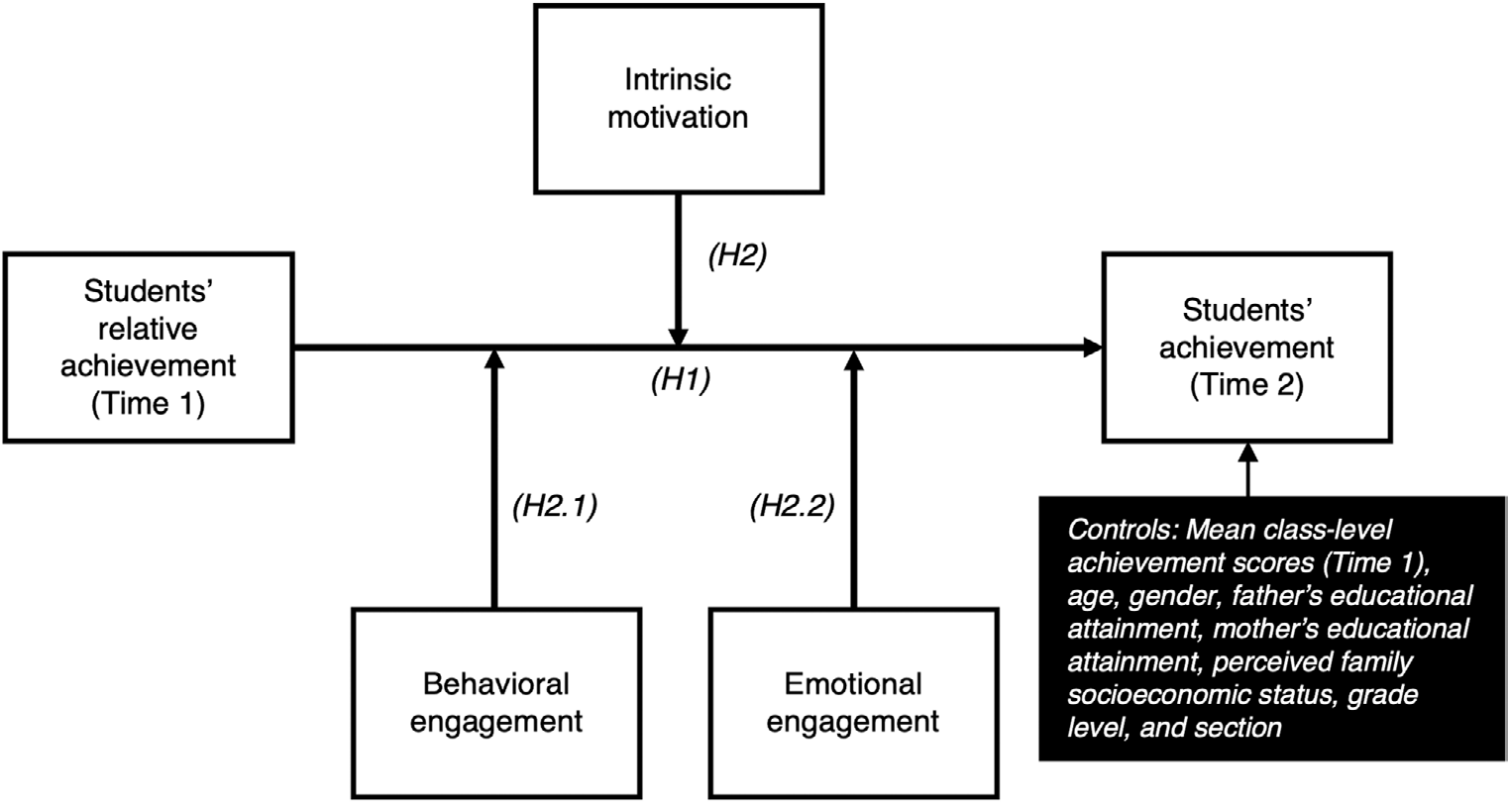
Conceptualizing achievement composition effect in L2 and individual‐level moderators.

We hypothesize that: (H1) Students' relative achievement at Time 1 will predict their achievement at Time 2, supporting the presence of ACE in L2 classrooms. (H2) Intrinsic motivation will moderate the relationship between relative achievement and subsequent achievement, with ACE being stronger among students with higher levels of: (H2a) intrinsic motivation to know, (H2b) intrinsic motivation to accomplish, and (H2c) intrinsic motivation to experience stimulation. (H3) Behavioural engagement will moderate the relationship between relative achievement and subsequent achievement, with ACE being stronger among students with higher levels of behavioural engagement. (H4) Emotional engagement will moderate the relationship between relative achievement and subsequent achievement, with ACE being stronger among students with higher levels of emotional engagement (see Figure 1).

## METHODS

### Participants and procedures

The data for this study were collected in a publicly funded secondary school in the Philippines. All procedures were conducted in accordance with the Ethics Committee's recommendations at the first and second authors' affiliations, following ethical approval. Informed Consent was sought from the students. We collected the data through a paper‐and‐pen survey at baseline (Time 1). Achievement data in English language learning T1 was also collected from the school. Eight weeks later (Time 2), achievement scores were collected again. The achievement scores were then paired to the respective students' respondent ID.

Thirty classrooms participated in this study (*n* = 796). The participants were from Grades 7 to 10, with 228, 180, 178 and 180 students from each grade, respectively. After excluding 30 students with incomplete achievement or self‐report data, the final sample size consisted of 766 students. The average number of students per class is 25.5. The mean age of the participants was 14.12 years old (*SD* = 1.52), and the sample was almost evenly split between boys and girls (*n* = 412 girls, 53.79%).

### Measures

#### Demographics

Demographic information (i.e. gender, age, father's educational attainment, mother's educational attainment, perceived family socioeconomic status, grade level and section) was collected through a demographic sheet.

#### Intrinsic motivation

The nine‐item Intrinsic Motivation (IM) subscale of the adapted Academic Motivation Scale (AMS; Guay et al., 2010; King & Caleon, 2021; Vallerand et al., 1992) was used to assess three components: IM to Know (e.g. “Because I feel happy and satisfied while learning new things.”), IM to Accomplish (e.g. “Because high school education allows me to feel satisfied when I work hard to excel in my studies.”), and IM to Experience Stimulation (e.g. “Because I really like going to school.”). The beginning sentence of the scale instruction was adjusted to “I study English because…” to assess students' intrinsic motivation to study English language learning. Each IM component was measured with three items that students responded to using the scale of 1 (“*strongly disagree*”) to 7 (“*strongly agree*”). In this study, the internal reliabilities of the IM to know, to accomplish, and to experience stimulation subscales are α = .73, α = .74 and α = .76, respectively.

#### Engagement

Student engagement in school was measured using the behavioural engagement (e.g. “When I'm in class, I participate in class discussions.”) and emotional engagement (e.g. “When I'm in class, I feel good.”) subscales of the Engagement and Disaffection Scale developed by Skinner, Kindermann, Connell, and Wellborn (2009) and Skinner, Kindermann, and Furrer (2009). Both subscales have five items, which were rated on a 4‐point scale (1 = “*strongly disagree*”, and 4 = “*strongly agree*”), with higher scores indicating a greater engagement. The Cronbach's alpha for the behavioural and emotional engagement subscales for this study were α = .80 and α = .79, respectively.

#### Achievement scores in English language learning

Secondary schools in the Philippines use a standards‐ and competency‐based grading system. Grades are weighted raw scores of the students' examinations and schoolwork (Department of Education, 2013, 2016). These achievement scores are composite or cumulative scores composed of assessment scores on cognitive tests (e.g. quizzes and examinations), classroom activities (e.g. recitations, presentations, group activities), and attendance. The academic year in the Philippine basic education system consists of four quarters, with achievement scores computed for each quarter. For this study, the third quarter (Time 1) and fourth quarter (Time 2) grades in English language learning were used. The average grade of the students in the 3rd quarter was 82.97 (*SD* = 5.74), and the average grade for the 4th quarter was 85.50 (*SD* = 6.06).

#### Student relative achievement

To assess compositional effects, we calculated each student's relative achievement at Time 1 by subtracting their classroom's mean achievement score from their individual score (see Marsh et al., 2023), with positive values indicating initial scores above the class average and negative values indicating scores below. This measure captures each student's achievement standing relative to their specific classroom peers, an important predictor in compositional effects research that accounts for the contextual influence of the classroom's achievement level on individual student outcomes.

### Data analysis

Prior to the main analyses, we used multiple imputation by chained equation (MICE; Azur et al., 2011) for all missing self‐report data. Only *n* = 37 participants had item‐level missing data (ranging from 5% to 32%). Aligned with the recommendations by Becker et al. (2022), Lüdtke et al. (2009), and Marsh et al. (2023), our analysis centred on disentangling individual and classroom compositional effects on achievement. Relative achievement at Time 1 was the key predictor representing ACE. As Marsh et al. (2023) emphasize, this group‐mean‐centred measure captures each student's achievement standing relative to classmates, making it well suited for assessing contextual influences.

A series of two‐level mixed‐effects linear regression models were estimated using the lmer function in R (R Core Team, 2016), nesting students (Level 1) within classrooms (Level 2). The null model revealed an ICC of .207, indicating that 20.7% of variance in Time 2 achievement was attributable to classroom‐level differences, justifying multilevel modelling (Gavin & Hofmann, 2002). Model 1 added student‐level demographic covariates (age, gender, parents' education, family SES). Model 2 introduced the class‐mean Time 1 achievement to assess contextual effects. Model 3 included relative achievement at Time 1, representing ACE. Models 4, 5, and 6 tested interactions of relative achievement with IM to Know, IM to Accomplish, and IM to Experience Stimulation, respectively. These moderated models aimed to examine (1) the contextual effect of relative Time 1 achievement on Time 2 achievement (i.e. ACE), and (2) the potential interactions where ACE can differ based on students' motivational orientations. Subsequently, behavioural engagement (Model 7) and emotional engagement (Model 8) were similarly tested as potential ACE moderators. This aligns with Marsh et al.'s (2023) emphasis on exploring individual‐level factors that may amplify or attenuate compositional influences (see also Becker et al., 2022). Significant interactions were further probed using simple slopes analysis and Johnson‐Neyman plots for visual representation.

## RESULTS

Table 1 includes all mixed‐effects models. The baseline Model 1 included student‐level covariates (gender, age, mothers' education, fathers' education, family SES). Gender emerged as a significant predictor, with girls scoring higher than boys on Time 2 achievement [*β* = 2.37, *SE* = .39, *t*(735.90) = 6.13, *p <* .001]. Mothers' education was also a significant predictor [*β* = .58, *SE* = .20, *t*(735.86) = 2.87, *p* = .004]. Model 2 added the classroom mean Time 1 achievement to account for the class‐aggregate effect. However, this aggregate classroom achievement did not significantly predict students' Time 2 individual achievement scores [*β* = .41, *SE* = .26, *t*(28.81) = 1.62, *p* = .117].

**TABLE 1:** Multilevel models examining the moderating role of age, behavioural engagement and emotional engagement on the influence of class‐aggregate achievement in Time 1 on student‐level achievement in Time 2.

	Model 1	Model 2	Model 3	Model 4	Model 5	Model 6	Model 7	Model 8
	*β* (*t*, SE, [95% CI])	*β* (*t*, SE, [95% CI])	*β* (*t*, SE, [95% CI])	*β* (*t*, SE, [95% CI])	*β* (*t*, SE, [95% CI])	*β* (*t*, SE, [95% CI])	*β* (*t*, SE, [95% CI])	*β* (*t*, SE, [95% CI])
Intercept	83.67***	50.15*	56.25*	55.23*	57.71*	54.28*	53.90*	54.61*
	(26.37, 3.17, [77.46: 89.89])	(2.40, 20.92, [9.15: 91.15])	(2.70, 20.80, [15.49: 97.02])	(2.66, 20.76, [14.55: 95.92])	(2.76, 20.89, [16.77: 98.65])	(2.63, 20.64, [13.83: 94.74])	(2.73, 19.76, [15.17: 92.63])	(2.70, 20.21, [15.00: 94.23])
Gender	2.37***	2.35***	.40	.41	.38	.42	.43	.43
	(6.13, .39, [1.61: 3.13])	(6.09, .39, [1.60: 3.11])	(1.40, .29, [−.16: .96])	(1.43, .29, [−.15: .97])	(1.33, .29, [−.18: .94])	(1.47, .28, [−.14: .97])	(1.53, .28, [−.12: .99])	(1.51, .28, [−.13: .99])
Age	−.35	−.39*	.00	.00	.01	.02	−.02	−.00
	(−1.81, .19, [−.73: .03])	(−2.03, .19, [−.77: −.01])	(.01, .15, [−.28: .29])	(.02, .15, [−.28: .29])	(.07, .15, [−.27: .30])	(.15, .14, [−.26: .30])	(−.16, .14, [−.30: .26])	(−.03, .14, [−.29: .28])
Mother's education	.58**	.58**	.08	.07	.07	.10	.05	.06
	(2.87, .20, [.18: .97])	(2.88, .20, [.19: .98])	(.55, .15, [−.20: .37])	(.50, .15, [−.21: .36])	(.49, .15, [−.21: .36])	(.66, .14, [−.19: .38])	(.35, .14, [−.23: .33])	(.40, .14, [−.23: .34])
Father's education	.26	.25	−.02	−.02	−.01	−.03	−.10	−.10
	(1.38, .19, [−.11: .62])	(1.35, .19, [−.11: .62])	(−.13, .13, [−.28: .25])	(−.16, .13, [−.28: .24])	(−.10, .13, [−.28: .25])	(−.24, .13, [−.29: .23])	(−.72, .13, [−.36: .17])	(−.76, .13, [−.37: .16])
SES	.04	.04	−.03	−.03	−.02	−.03	−.05	−.04
	(.35, .11, [−.18: .26])	(.31, .11, [−.18: .25])	(−.37, .08, [−.19: .13])	(−.41, .08, [−.19: .13])	(−.27, .08, [−.18: .14])	(−.40, .08, [−.19: .13])	(−.65, .08, [−.21: .11])	(−.52, .08, [−.20: .12])
T1 achievement		.41	.35	.35	.34	.34	.35	.35
		(1.62, .26, [−.09: .91])	(1.37, .25, [−.15: .84])	(1.40, .25, [−.14: .85])	(1.34, .25, [−.16: .83])	(1.36, .25, [−.15: .83])	(1.46, .24, [−.12: .82])	(1.43, .25, [−.13: .83])
T1 relative achievement			.71***	.85***	.69***	.16	.32*	.29*
			(26.55, .03, [.66: .76])	(6.35, .13, [.59: 1.11])	(4.35, .16, [.38: 1.00])	(1.02, .16, [−.15: .47])	(2.55, .13, [.07: .57])	(2.29, .13, [.04: .54])
Intrinsic motivation to know (IMTK)				.09				
				(.63, .14, [−.19: .37])				
T1 relative achievement × IMTK				−.03				
				(−1.04, .03, [−.08: .02])				
Intrinsic motivation to accomplish (IMTA)					−.20			
					(−1.12, .18, [−.55: .15])			
T1 relative achievement × IMTA					.00			
					(.14, .03, [−.06: .07])			
Intrinsic motivation to experience stimulation (IMES)						.37*		
						(2.35, .16, [.06: .67])		
T1 relative achievement × IMES						.10***		
						(3.45, .03, [.04: .16])		
Behavioural engagement (BE)							.95***	
							(3.68, .26, [.44: 1.46])	
T1 relative achievement × BE							.12**	
							(2.84, .04, [.04: .21])	
Emotional engagement (EE)								.59*
								(2.30, .26, [.09: 1.09])
T1 relative achievement × EE								.14**
								(3.30, .04, [.06: .22])
N	766	766	766	766	766	766	766	766
N (Classes)	30	30	30	30	30	30	30	30
ICC/Adj. ICC	.227/.213	.218/.201	.367/.216	.365/.214	.368/.217	.366/.212	.347/.197	.356/.205
AIC	4785.18	4785.49	4298.69	4308.51	4307.79	4295.91	4286.27	4292.71
BIC	4822.31	4827.26	4345.10	4364.20	4363.48	4351.61	4341.96	4348.40
R2 (fixed)	.06	.08	.41	.41	.41	.42	.43	.43
R2 (total)	.28	.28	.63	.63	.63	.63	.63	.63

***p < .001; **p < .01; *p < .05.

The key predictor of interest, students' relative Time 1 achievement, was introduced in Model 3. As hypothesized, relative achievement significantly predicted Time 2 achievement [*β* = .71, *SE* = .03, *t*(732.83) = 26.55, *p* < .001], providing evidence for the achievement composition effect (ACE).

Models 4 and 5 examined if ACE varied based on students' IM to Know and IM to Accomplish, respectively, by including interactions between relative Time 1 achievement and these motivational orientations. Neither the main effects of IM to Know [*β* = .09, *SE* = .14, *t*(735.17) = .63, *p* = .53] and IM to Accomplish [*β* = −.20, *SE* = .18, *t*(734.16) = −1.12, *p* = .27] nor their interactions with relative achievement [IM to Know: *β* = −.03, *SE* = .03, *t*(735.02) = −1.04, *p* = .30; IM to Accomplish: *β* = .01, *SE* = .03, *t*(731.70) = .14, *p* = .89] were significant, suggesting that these motivational orientations did not moderate ACE.

**FIGURE 2:**
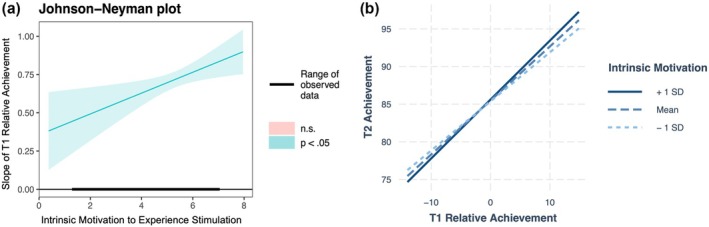
Johnson‐Neyman (left) and simple slopes (right) plots showing how the slope or correlation between T1 class relative achievement and T2 student‐level achievement strengthens as Intrinsic Motivation to Experience Stimulation increases.

Model 6 examined if ACE varied based on students' IM to Experience Stimulation by including an interaction between relative Time 1 achievement and IM to Experience Stimulation. The main effect of IM to Experience Stimulation was significant [*β* = .37, *SE* = .16, *t*(737.41) = 2.35, *p* = .02], indicating that students with higher levels of this motivational orientation tended to have higher Time 2 achievement scores. Importantly, the interaction term was also significant [*β* = .10, *SE* = .03, *t*(731.98) = 3.45, *p* < .001], suggesting that ACE was stronger for students with higher levels of IM to Experience Stimulation. The Johnson‐Neyman (JN) technique (Figure 2) revealed that the effect of relative achievement on Time 2 achievement was significant when IM to Experience Stimulation scores were outside the interval [−10.79, .95], indicating that ACE was significant across all observed levels of this motivational orientation [1.33, 7.00]. Simple slopes analysis showed that the effect of relative achievement on Time 2 achievement increased from *β* = .60 at low levels (−1 SD) to *β* = .80 at high levels (+1 SD) of IM to Experience Stimulation.

**FIGURE 3:**
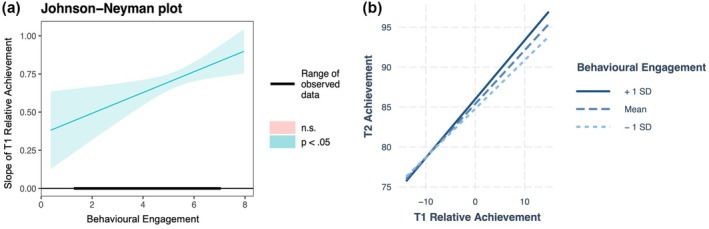
Johnson‐Neyman (left) and simple slopes (right) plots showing how the slope or correlation between T1 class relative achievement and T2 student‐level achievement strengthens as behavioural engagement increases.

**FIGURE 4:**
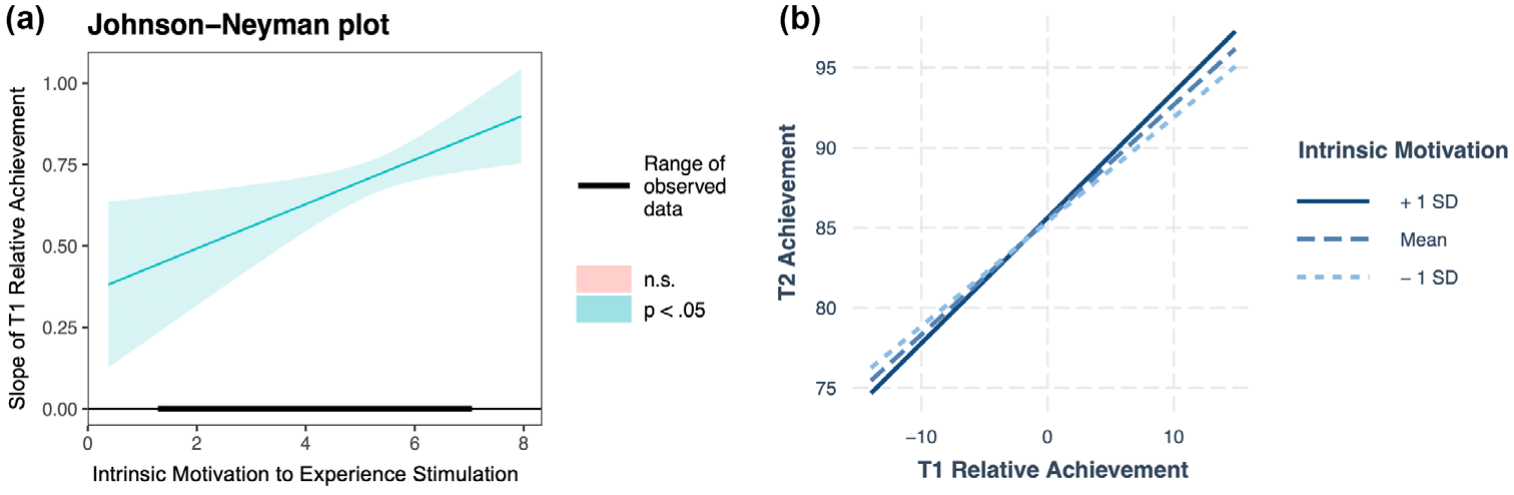
Johnson‐Neyman (left) and simple slopes (right) plots showing how the slope or correlation between T1 class relative achievement and T2 student‐level achievement strengthens as emotional engagement increases.

Models 7 and 8 tested behavioural engagement and emotional engagement as potential moderators of ACE, respectively. In both models, the main effects of behavioural engagement [*β* = .95, *SE* = .26, *t*(739.46) = 3.68, *p* < .001] and emotional engagement [*β* = .59, *SE* = .26, *t*(741.23) = 2.30, *p* = .02] were significant, indicating that students with higher levels of these engagement factors tended to have higher Time 2 achievement scores. Moreover, the interactions between relative achievement and behavioural engagement [*β* = .12, *SE* = .04, *t*(730.88) = 2.84, *p* = .01] and emotional engagement [*β* = .14, *SE* = .04, *t*(730.18) = 3.30, *p* < .001] were also significant, suggesting that ACE was stronger for students with higher levels of engagement. JN analyses (Figures 3 and 4) revealed that ACE was significant across all observed levels of behavioural engagement [1.20, 4.00] and emotional engagement [1.00, 4.00]. Simple slopes analyses showed that the effect of relative achievement on Time 2 achievement increased from *β* = .60 at low levels (−1 SD) to *β* = .74 at high levels (+1 SD) of behavioural engagement, and from *β* = .61 at low levels to *β* = .77 at high levels of emotional engagement.

## DISCUSSION

The present study provides empirical evidence that the achievement composition effect (ACE) extends to L2 learning contexts among secondary school students from the Philippines. Results show that students' relative achievement, compared to their classroom peers, emerged as a robust predictor of their later achievement (supporting H1). Specifically, students who scored above their class average in English at Time 1 showed significantly higher English achievement at Time 2, even after controlling for class means and other demographics. Peer achievement levels comprising a student's classroom environment appear to be an influential force shaping their language development over time. This finding substantiates the first empirical evidence demonstrating ACE's impact in L2 instructional settings, extending its well‐established influence from other specific learning domains (Becker et al., 2022; Hanushek et al., 2003; Marsh et al., 2023) to language acquisition contexts.

### Achievement composition effect on L2 learning

Broadly, the findings corroborate previous studies demonstrating that class‐average achievement is positively associated with individual achievement gains across various learning domains (Becker et al., 2022; Getenet, 2023; Hanushek et al., 2003). Our results extend the work of Mashburn et al. (2009) on classroom peer effects in language development and contribute to the growing body of literature on ACE in school achievement (Gottfried, 2014; Steenberghs et al., 2021; Vu et al., 2022).

Based on SDT (Ryan & Deci, 2017) and SSMMD (Skinner, Kindermann, Connell, & Wellborn, 2009; Skinner, Kindermann, & Furrer, 2009), ACE can be understood as a manifestation of the social context's influence on students' basic psychological needs satisfaction, motivation, and achievement. Being surrounded by high‐achieving peers can facilitate L2 learning by fostering supportive classroom climates that boost students' sense of competence, autonomy, and relatedness, leading to greater L2 learning motivation and engagement (Ryan & Deci, 2017; Skinner, Kindermann, Connell, & Wellborn, 2009; Skinner, Kindermann, & Furrer, 2009). When most classmates display higher L2 abilities, students may perceive L2 skills as a class norm and internalize the motivation to develop their proficiencies (see Eccles & Wigfield, 2020; e.g., Mendoza & King, 2020; King & Mendoza, 2020).

Our findings demonstrate how this process operates across achievement levels. Consider, for example, two high‐achieving students in our sample who scored one standard deviation above their respective class means at Time 1. Those in classrooms with above‐average mean achievement (M = 84.5) showed greater improvement (3.1 points) compared to their counterparts in classrooms with below‐average mean achievement (M = 82.5, improvement = 2.3 points). This pattern persisted despite these students occupying similar relative positions (+SD above the class mean) within their respective classes. Such results suggest that while previous individual achievement strongly predicts future performance, the achievement composition of the classroom provides an additional, distinct effect on student achievement, potentially through enhanced opportunities for need satisfaction and engagement in higher‐achieving classroom environments.

Related theories also lend support to how ACE may manifest in L2 classrooms. Social learning theory (Bandura, 1986) suggests that students can acquire L2 competencies by observing and modelling higher‐achieving peers. The peer group provides a vital source of information exchange, examples to emulate, and socialization around language use (Sato & Ballinger, 2016). Learning opportunities arise through interactions and observations of more capable peers, creating opportunities for feedback, clarification, and authentic communication, facilitating L2 learning, and raising students' self‐efficacy (Philp et al., 2014; Schunk & DiBenedetto, 2020). This is particularly relevant, especially among younger learners (Bandura, 1997). Secondary school students may be more receptive to the behaviour and performance of their high‐achieving peers, leading to greater motivation and improved achievement. Previous studies also highlighted peers' roles in establishing classroom norms, academic behaviours, and even academic achievement (Laninga‐Wijnen et al., 2018; Steinberg, 2011; Urdan & Schoenfelder, 2006). It is plausible that younger students, when interacting with peers and comparing their academic behaviours, are more easily influenced by their peers' actions and peer influence (see Marsh et al., 2023; McCormick & Cappella, 2015; Stäbler et al., 2017; Steinberg, 2011).

While L2 achievement is influenced by many factors (Dörnyei & Ryan, 2015; Ortega, 2009), our results underscore the importance of considering the achievement composition of L2 classrooms. Given the inherent diversity of achievement levels within classrooms, understanding the unfolding of these compositional effects is crucial for designing optimal L2 learning environments. Tracking policies, strategic classroom assignments, and efforts to foster students' intrinsic motivation and engagement may present potential avenues to leverage positive ACE while mitigating negative dynamics arising from unfavourable peer compositions (Becker et al., 2022; Fortuin et al., 2015; Marsh et al., 2023).

### Increased intrinsic motivation and engagement strengthens ACE

Our results show that the strength of ACE in L2 classrooms is conditional on students' levels of intrinsic motivation to experience stimulation (supporting H2c), behavioural engagement (supporting H3), and emotional engagement (supporting H4). Specifically, the positive effect of class‐level achievement at Time 1 on student‐level achievement at Time 2 is moderated by individual differences in motivation and engagement factors, with higher levels of these factors amplifying the impact of peer achievement on individual achievement.

The emergence of intrinsic motivation to experience stimulation as a significant moderator of ACE, while intrinsic motivation to know and to accomplish did not (rejecting H2a and H2b), highlights the differential role of motivational orientations in peer influence susceptibility (Noels et al., 2019). Students motivated by the sensory pleasure, aesthetic experiences, and excitement associated with learning (Vallerand et al., 1992, 1993) may be more receptive to high‐achieving peers' engaging behaviours (Shin & Ryan, 2014; Ushioda, 2011). Consequently, the social transmission of achievement‐related attitudes and behaviours may be more pronounced among these students (Burgess et al., 2018; Parr & Townsend, 2002).

One possible explanation for this moderating effect is that intrinsic motivation to experience stimulation may be more closely tied to the social and affective aspects of learning, which are critical in L2 acquisition (Dewaele & MacIntyre, 2014; Dörnyei, 2019). Students motivated by the inherent enjoyment and excitement of learning may be more responsive to the positive emotional climate created by their high‐achieving peers (see Moskowitz & Dewaele, 2021), thereby amplifying the social contagion of achievement. In contrast, intrinsic motivation to know and to accomplish, which focuses more on the inherent satisfaction derived from learning and mastering new skills (Vallerand et al., 1992, 1993), may be less susceptible to the influence of peer achievement (Noels et al., 2019; Oga‐Baldwin, 2019).

Similarly, significant moderating effects of behavioural and emotional engagement suggest that students who actively participate in classroom activities and experience positive emotions are more receptive to the learning behaviours and attitudes of their high‐achieving peers, thus amplifying the impact of peer achievement on individual performance (Fredricks et al., 2004; Furrer & Skinner, 2003; Skinner, Kindermann, Connell, & Wellborn, 2009; Skinner, Kindermann, & Furrer, 2009). This is aligned with previous work demonstrating the facilitative effect of engagement on academic outcomes and susceptibility to classroom contexts (Furrer et al., 2014; Skinner et al., 2008; Vollet et al., 2017). Highly engaged students likely experience greater motivational benefits from higher‐achieving classroom compositions, such as increased self‐efficacy, psychological need satisfaction, and resilience stemming from positive peer models (Blansky et al., 2013; Gremmen et al., 2017; Ruzek et al., 2016; Schunk & DiBenedetto, 2020; Shin & Ryan, 2014; Wentzel & Caldwell, 1997). Conversely, disengaged students could be less impacted by peer composition, positive or negative (Vollet et al., 2017). This suggests that engaged students are more attuned to social cues (Wentzel, 2009) and better positioned to absorb motivational influences from high‐achieving peers through processes like modelling (Schunk & DiBenedetto, 2020), socialization, and internalized classroom norms (e.g., Mendoza & King, 2020, 2021).

The conditional effects of intrinsic motivation to experience stimulation and engagement on ACE can also be understood through how the person's characteristics align with or fit the environment (e.g., Pawlowska et al., 2014; van Vianen, 2018). Students whose motivational orientations and engagement levels are congruent with the positive emotional climate and active learning behaviours fostered by high‐achieving peers may be more likely to benefit from the social transmission of achievement. In other words, the impact of peer achievement on individual achievement may be optimized when there is a match between students' motivational and engagement profiles and the classroom environment shaped by high‐achieving peers (Eccles & Roeser, 2011; Juvonen et al., 2012).

These findings highlight the importance of considering both peer achievement levels and individual differences in students' motivation and engagement within the context of L2 acquisition. By creating classroom environments that nurture intrinsic motivation to experience stimulation and foster behavioural and emotional engagement, educators can harness the motivational benefits of social contagion and optimize the impact of peer achievement on individual achievement (Mercer & Dörnyei, 2020; Niemiec & Ryan, 2009; Ushioda, 2011). In L2 classrooms, this can be achieved through engaging language learning activities, the provision of choice and autonomy support in L2 tasks, and a supportive classroom climate that encourages active participation and peer interaction (Mercer & Dörnyei, 2020; Zadorozhnyy & Lee, 2024).

### Study limitations and directions for future research

The present study has limitations that should be considered. First, the reliance on self‐reported data may have introduced response biases. Future studies could employ additional measures, such as observational data, teacher ratings, or experience sampling methods, to provide a more comprehensive understanding of motivation and engagement's moderating influence on ACE (e.g. Aunola et al., 2013; Pekrun & Bühner, 2014). Second, while the study examined classroom achievement compositions using relative achievement test scores, other potential peer characteristics and mechanisms that could impact ACE were not investigated. Future research could integrate classroom observations, peer nominations and social network analysis to elucidate nuanced peer processes (see Madill et al., 2014; Raufelder et al., 2013). Finally, the generalizability of the findings to other academic domains, developmental stages and educational contexts remains to be explored. Future studies should investigate ACE across varied age ranges and instructional settings, particularly in L2 learning contexts (Ballinger et al., 2017; Henry & Thorsen, 2018).

Despite these limitations, the present study offers theoretical and practical insights. The results provide an integrative perspective synthesizing the literature on achievement composition, intrinsic motivation, engagement, and language learning to understand how these constructs interact within L2 classrooms. The findings also offer practical insights, affirming that ACE operates in formal L2 contexts, but its impact depends on students' motivational orientations and engagement levels. This highlights how social and individual factors synergistically shape academic trajectories (Eccles & Wigfield, 2020; Haerens, 2020).

Further, the findings extend previous research on the achievement composition effect in L2 classrooms, showing that students with higher intrinsic motivation to experience stimulation and those with higher engagement levels benefit more from high‐achieving peers (Becker et al., 2022; Lüdtke et al., 2009; Marsh et al., 2023). This aligns with the perspective that peers exert greater influence during early developmental stages (Erikson et al., 2021; Harris & Vazire, 2016) and underscores the role of motivation and engagement in shaping academic outcomes (Martin & Dowson, 2009; Wentzel & Muenks, 2016). The moderating effects of intrinsic motivation and engagement highlight the importance of considering individual differences when investigating contextual effects on achievement (Haerens, 2020; King & McInerney, 2019). Particularly among secondary school students in L2 classrooms, classroom relative achievement in language learning is influenced by factors such as intrinsic motivation and behavioural and emotional engagement.

## CONCLUSION

This study extends our understanding of achievement composition effect (ACE) within second language learning contexts. This reveals not just the presence of compositional effects but also the crucial moderating roles of specific motivational and engagement factors that amplify these effects. The findings demonstrate that classroom achievement composition functions more than a contextual variable in that it serves as a dynamic factor catalysing individual psychological resources to shape L2 learning trajectories. This interaction suggests that the “rising tide” metaphor extends beyond mere peer effects to encompass a complex interplay between social‐contextual and individual psychological factors in L2 acquisition. The emergence of intrinsic motivation to experience stimulation, behavioural engagement, and emotional engagement as significant moderators of ACE points to distinct psychological pathways through which the social contagion of achievement operates. This suggests that creating optimal L2 learning environments requires not just attention to classroom composition but also deliberate cultivation of these psychological resources that make students more receptive to positive peer influences. These insights extend beyond L2 classrooms to our broader understanding of how social and individual factors combine to create effective learning environments, calling for a more nuanced approach to classroom composition that considers both the social ecology of achievement and the psychological characteristics that help students benefit from it. These findings open new avenues for research exploring how classroom composition and motivational factors might optimize learning across various educational contexts, potentially informing educational policies and practices that can better harness the power of peer effects while nurturing the individual psychological qualities that make these effects most beneficial.

## AUTHOR CONTRIBUTIONS

**Norman B. Mendoza:** Conceptualization; investigation; methodology; validation; visualization; writing – review and editing; writing – original draft; formal analysis; supervision; software; data curation. **Artem Zadorozhnyy:** Conceptualization; methodology; validation; writing – original draft; writing – review and editing; project administration; software. **John Ian Wilzon T. Dizon:** Conceptualization; writing – original draft; methodology; project administration; writing – review and editing; data curation.

## FUNDING INFORMATION

During the conduct of this research, Dr. Norman Mendoza has been supported by the Hong Kong Research Grants Council (RGC) Postdoctoral Fellowship Scheme, Hong Kong SAR, China. Grant Number: PDFS 2223‐8H07.

## CONFLICT OF INTEREST STATEMENT

The authors declare no conflicts of interest.

## ETHICS STATEMENT

The Human Research Ethics Committee of the first author's affiliated university approved the methods of the study.

## CONSENT TO PARTICIPATE

Informed assent forms were sought from the students. The parents/guardians also gave their informed consent.

## CONSENT FOR PUBLICATION

All legal guardians and the participants themselves, including the school where they were enrolled in, gave their consent/assent to publish manuscripts that used their data.

## Data Availability

The data that support the findings of this study are available from the corresponding author upon reasonable request.
